# Glutamine supports the protection of tissue cells against the damage caused by cholesterol-dependent cytolysins from pathogenic bacteria

**DOI:** 10.1371/journal.pone.0219275

**Published:** 2020-03-12

**Authors:** Matthew L. Turner, Sian E. Owens, I. Martin Sheldon

**Affiliations:** Institute of Life Science, Swansea University Medical School, Swansea University, Swansea, Wales, United Kingdom; Laurentian University, CANADA

## Abstract

Pathogenic bacteria often damage tissues by secreting toxins that form pores in cell membranes, and the most common pore-forming toxins are cholesterol-dependent cytolysins. During bacterial infections, glutamine becomes a conditionally essential amino acid, and glutamine is an important nutrient for immune cells. However, the role of glutamine in protecting tissue cells against pore-forming toxins is unclear. Here we tested the hypothesis that glutamine supports the protection of tissue cells against the damage caused by cholesterol-dependent cytolysins. Stromal and epithelial cells were sensitive to damage by the cholesterol-dependent cytolysins, pyolysin and streptolysin O, as determined by leakage of potassium and lactate dehydrogenase from cells, and reduced cell viability. However, glutamine deprivation increased the leakage of lactate dehydrogenase and reduced the viability of cells challenged with cholesterol-dependent cytolysins. Without glutamine, stromal cells challenged with pyolysin leaked lactate dehydrogenase (control vs. pyolysin, 2.6 ± 0.6 vs. 34.4 ± 4.5 AU, n = 12), which was more than three-fold the leakage from cells supplied with 2 mM glutamine (control vs. pyolysin, 2.2 ± 0.3 vs. 9.4 ± 1.0 AU). Glutamine cytoprotection did not depend on glutaminolysis, replenishing the Krebs cycle via succinate, changes in cellular cholesterol, or regulators of cell metabolism (AMPK and mTOR). In conclusion, although the mechanism remains elusive, we found that glutamine supports the protection of tissue cells against the damage caused by cholesterol-dependent cytolysins from pathogenic bacteria.

## Introduction

Animals defend themselves against bacterial infections using the complimentary strategies of resistance and tolerance [1–3]. Resistance is the ability to limit the pathogen burden, usually by employing the immune system to kill bacteria. Tolerance is the ability to limit the severity of disease caused by the pathogen burden, usually by limiting the damage caused by bacteria. Bacteria often damage tissue cells by secreting toxins that form pores in the cell membrane, and the most common pore-forming toxins are cholesterol-dependent cytolysins [4–7]. During bacterial infections, the cells of the immune system use glutamine as a key nutrient to support inflammatory responses [8–10]. However, the role of glutamine in protecting tissue cells against the damage caused by cholesterol-dependent cytolysins is unclear.

Cholesterol-dependent cytolysins include pyolysin secreted by *Trueperella pyogenes*, which causes purulent infections in cattle and swine, such as postpartum uterine disease and abscesses, and streptolysin O (SLO) secreted by beta-hemolytic group A *Streptococci*, which causes pharyngitis and impetigo in children [11–14]. These cytolysins bind cholesterol-rich areas in tissue cell membranes, where they form 30 nm diameter pores. The membrane pores lead to leakage of potassium ions from cells within minutes, and further cell damage is evidenced by leakage of proteins, such as lactate dehydrogenase (LDH), from the cytoplasm and ultimately cell death [6, 15]. Tissue cells try to protect themselves against the damage by activating stress responses and transitioning to a quiescent metabolic state [5, 6]. However, the role of metabolism in protecting cells against cholesterol-dependent cytolysins is unknown. An intriguing observation is that the metabolic stress of lactation in dairy cattle increases the risk of postpartum uterine disease associated with *T*. *pyogenes* [16–19], probably by impairing the ability of the endometrial tissue to tolerate the presence of bacteria [20]. We therefore propose that the availability of nutrients might affect the ability of tissue cells to protect themselves against cholesterol-dependent cytolysins.

Cells use glucose and glutamine to supply most of their energy [21–23]. The enzymes of the glycolysis pathway convert glucose to pyruvate to feed the Krebs cycle, whilst glutaminase converts glutamine to glutamate to replenish the Krebs cycle [9, 24]. Glutamine is an abundant non-essential amino acid, with about 0.7 mM glutamine in human peripheral plasma and 0.25 mM in bovine plasma [8, 25]. However, glutamine becomes a conditionally essential amino acid after injury or infection, and glutamine fosters immune cell inflammatory responses [8, 9, 26, 27]. As glutamine is a key nutrient, our aim was to test the hypothesis that glutamine supports the protection of tissue cells against the damage caused by cholesterol-dependent cytolysins. To test our hypothesis we manipulated the supply of glutamine in the culture media and examined the effects on cell viability and pore formation in both stromal and epithelial cells that were challenged with pyolysin and streptolysin O.

## Results

### Pyolysin damages stromal cells

**Fig 1:**
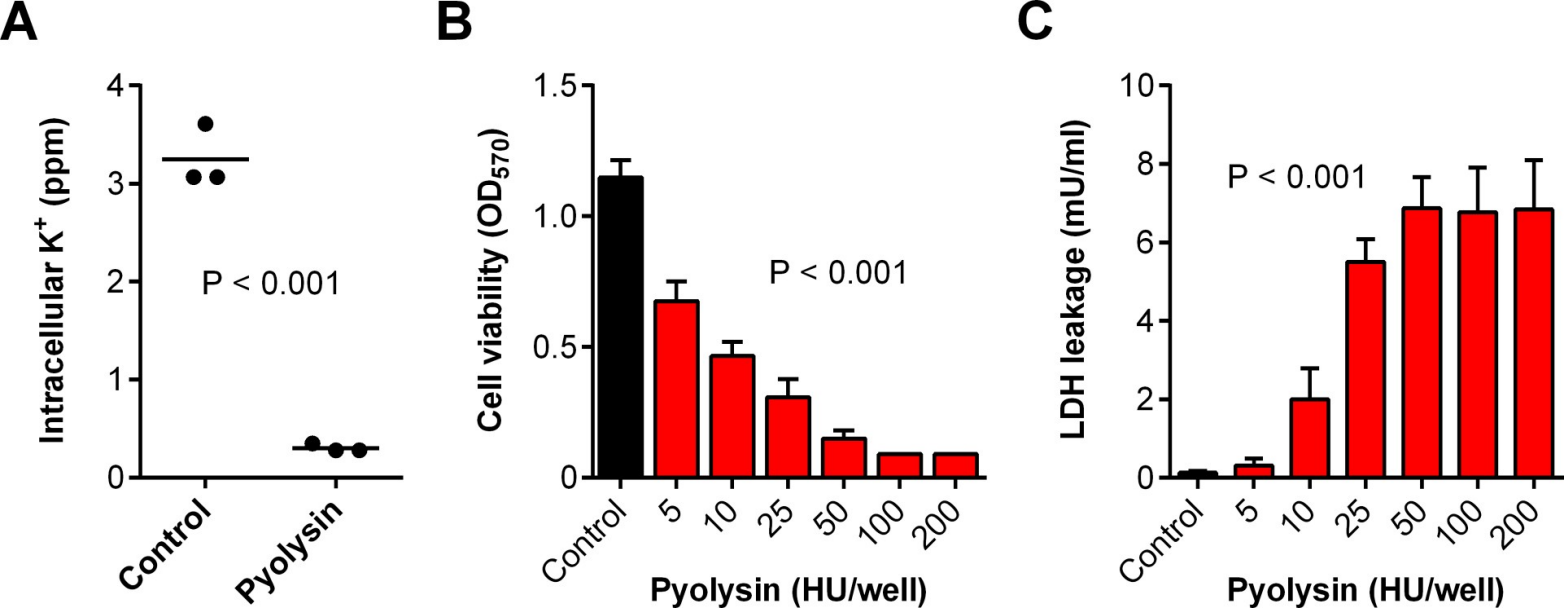
Cytolytic activity of pyolysin in stromal cells. (A) Primary bovine endometrial stromal cells were challenged for 5 min with control serum-free medium (•) or medium containing pyolysin (•), and potassium was measured in cell lysates. Data are presented using cells isolated from 3 animals and the horizontal line represents the mean; data were analyzed by t-test. (B, C) Stromal cells were challenged for 2 h with control serum-free medium (∎) or medium with the indicated concentrations of pyolysin (∎); cell viability was determined by MTT assay (B) and LDH leakage evaluated by measuring LDH in the cell supernatants (C). Data are presented as mean (SEM) using cells from 4 animals; data were analyzed by ANOVA and P values are reported.

We isolated primary bovine endometrial stromal cells from uteri collected from cows after slaughter, as described previously [14, 28, 29]. We used pyolysin to study cytoprotection because bovine endometrial stromal cells are a principal target for pyolysin [14]; and, unlike other cholesterol-dependent cytolysins, pyolysin does not require thiol-activation *in vitro* [13]. Pyolysin formed pores in the stromal cells, as determined by the loss of intracellular potassium within 5 min (Fig 1A). Furthermore, a 2 h challenge with pyolysin damaged the stromal cells, as determined by reduced cell viability (Fig 1B) and leakage of lactate dehydrogenase (LDH) from the cytosol into cell supernatants (Fig 1C). We chose a 2 h pyolysin challenge based on previous kinetic studies where 50% of endometrial stromal cells were perforated after 2 h [14]. Furthermore, the 2 h challenge reduces the likelihood of confounding cell protection with immune responses to the cytolysins, which are usually evident in immune cells after 2 h of challenge with cholesterol-dependent cytolysins [30].

### Glutamine supports stromal cell protection against pyolysin

**Fig 2:**
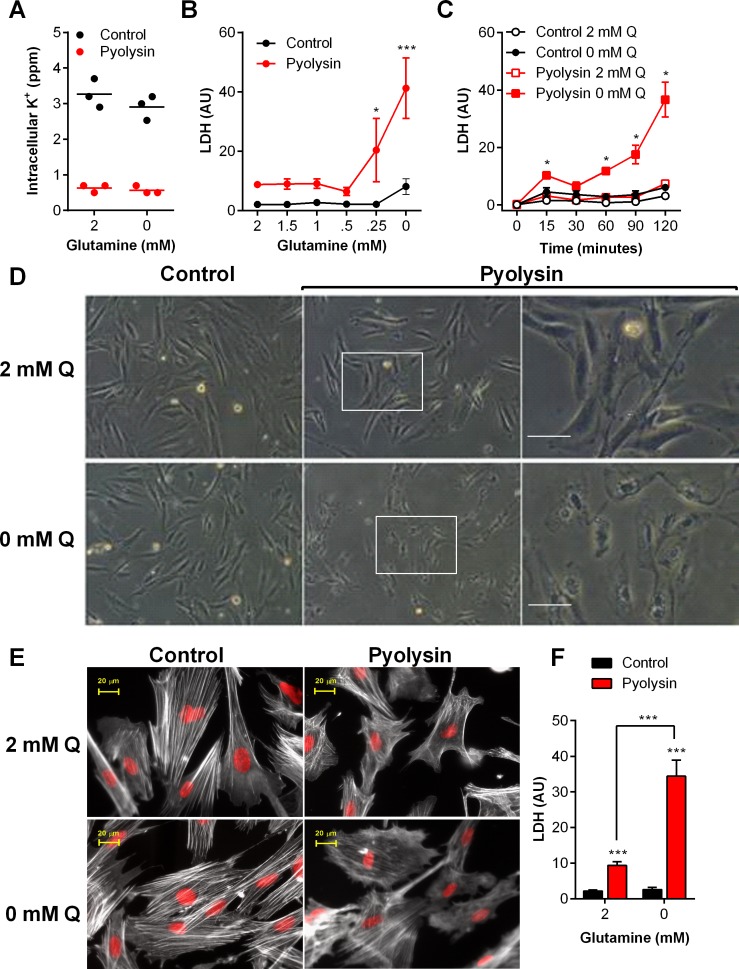
Glutamine is cytoprotective against pyolysin in stromal cells. (A) Bovine endometrial stromal cells were cultured for 24 h in medium containing 2 mM glutamine or without glutamine, and challenged for 5 min with control medium (•) or pyolysin (•). Intracellular potassium was determined by flame photometry. Data are from cells isolated from 3 animals, with a horizontal line indicating the mean. (B) Cells were cultured in medium containing the indicated concentrations of glutamine for 24 h, and challenged for 2 h with control medium (•) or 10 HU pyolysin (•), and LDH measured in supernatants and normalized to cellular DNA in the control challenge. Data are mean (SEM) of cells isolated from 4 animals, analyzed by ANOVA with Dunnett’s post hoc test; values differ from 2 mM glutamine pyolysin challenge, *** P < 0.001, * P < 0.05. (C) Stromal cells were cultured with 2 mM glutamine (Q, open symbols) or without glutamine (0 mM Q, filled symbols) for 24 h, and challenged for the indicated times with control medium or pyolysin; LDH leakage was measured in supernatants and normalized to cellular DNA in the control challenge. Data are mean (SEM) of cells isolated from 4 animals; analyzed by ANOVA with Dunnett’s post hoc test; values differ from 2 mM glutamine pyolysin challenge, * P < 0.05. (D) Stromal cells were cultured with glutamine (2 mM Q) or without glutamine (0 mM Q) for 24 h, and challenged for 2 h with control medium or pyolysin. Transmitted light micrographs of cells were captured at the end of the experiment; right column represents magnification of boxed areas from middle column; scale bar 10 μm; images are representative of cells from 4 animals. (E) Cells were also stained with Alexa Fluor 555-conjugated phalloidin to visualize F-actin (white) and fluorescent microscope images collected; nuclei are red; scale bars are 20 μm; images are representative of cells from 4 animals. (F) Cells were cultured for 24 h in medium containing 2 mM glutamine or without glutamine, and challenged for 2 h with control medium (∎) or 10 HU pyolysin (∎), and LDH measured in supernatants and normalized to cellular DNA in the control challenge. Data are mean (SEM) of cells isolated from 12 animals, analyzed by ANOVA with Bonferroni post hoc test; values differ from 2 mM glutamine pyolysin challenge, *** P <0.001.

Primary bovine endometrial stromal cells are usually cultured in media containing 2 mM glutamine [14, 28, 29], which is eight fold higher than the plasma concentration of glutamine in cows [25]. To examine if the availability of glutamine affected cytoprotection against pyolysin, we cultured stromal cells for 24 h in serum-free media containing an excess of glucose (11.1 mM) with a range of concentrations of glutamine (0 to 2 mM), and then challenged the cells for 2 h with control medium or 10 HU/well pyolysin. We used serum-free medium because cholesterol in serum might bind cholesterol-dependent cytolysins, and to limit glutamine-dependent differences in cell growth. Irrespective of glutamine availability, pyolysin caused pore formation, as determined by loss of intracellular potassium within 5 min (Fig 2A; two-way ANOVA, n = 3 animals, P < 0.001). We next evaluated the effect of glutamine on pyolysin-induced cell damage by determining the leakage of LDH into cell supernatants. We did not use the mitochondrial-dependent MTT assay for cell viability here because differences in glutamine availability affect cell growth and mitochondrial function [8]. Furthermore, to account for differences in cell growth, we measured cellular DNA at the end of each experiment and normalized the leakage of LDH into cell supernatants using the cellular DNA in the control challenge. Limiting the availability of glutamine increased the accumulation of LDH in supernatants when cells were challenged with pyolysin (Fig 2B; two-way ANOVA; n = 4 animals, P < 0.001). The increased leakage of LDH in stromal cells cultured without glutamine, compared with 2 mM glutamine, was evident from 15 min after pyolysin challenge (Fig 2C).

When cells were examined by light microscopy, cells cultured with 2 mM glutamine and challenged with pyolysin showed some damage but usually maintained defined cell boundaries, whereas most cells were misshapen and the cytoplasm and nuclei condensed if they were deprived of glutamine and challenged with pyolysin (Fig 2D). Staining the actin cytoskeleton with Alexa Fluor 555-conjugated phalloidin also showed that when challenged with pyolysin the cytoskeletal was more diffuse and disorganized, with less well defined actin fibers, and the cell shape was more disrupted in cells deprived of glutamine than cells cultured with glutamine (Fig 2E).

As primary cells often vary in their biological response, we verified our observation that glutamine helped protect cells against pyolysin using stromal cells isolated from 12 independent animals. The primary bovine endometrial stromal cells cultured without glutamine leaked more than three times the amount of LDH compared with cells cultured in medium containing 2 mM glutamine (Fig 2F).

**Fig 3:**
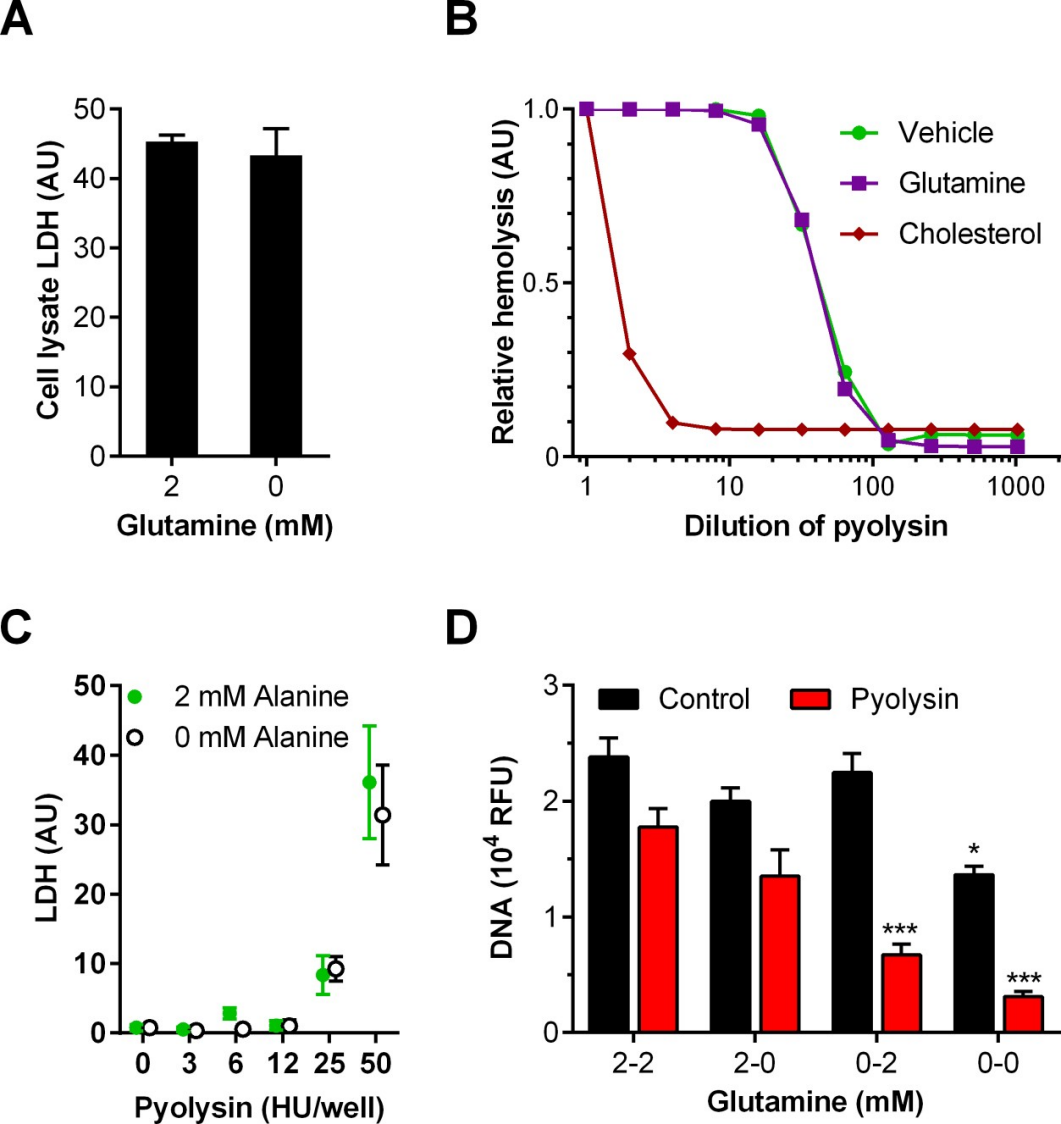
Glutamine is cytoprotective against pyolysin in stromal cells but does not alter intracellular LDH or bind pyolysin. (A) Whole cell lysates were collected after 24 h treatment with 2 mM or no glutamine, and intracellular LDH abundance measured, and normalized to cellular DNA. Data are mean (SEM) of cells isolated from 4 animals. (B) Red blood cells were challenged with serial dilutions of pyolysin, after a prior 1 h incubation of the pyolysin with vehicle (•), 2 mM glutamine (∎), or 1 μM cholesterol (◆). Hemolysis data are presented as mean of 2 experiments, with 2 replicates per treatment. (C) Bovine endometrial stromal cells were treated in glutamine-free medium containing no alanine (○) or 2 mM alanine (•) for 24 h prior to challenge with control vehicle (0) or the indicated concentrations of pyolysin, and the leakage of LDH from cells was measured in cell supernatants and normalized to cellular DNA in control vehicle. Data are presented as mean (SEM) from 4 animals. (D) Cells were cultured in serum-free medium containing 2 mM glutamine (2) or without glutamine (0) for 24 h before challenge with control medium (∎) or pyolysin (∎) for 2 h. The media were then replenished with medium containing 2 mM glutamine (2) or without glutamine (0) for a further 24 h and cellular DNA measured. Data are mean (SEM) from cells isolated from 4 animals, and analyzed by ANOVA and Bonferroni post hoc test; values differ from 2–2 within challenge group, * P < 0.05, *** P < 0.001.

We examined the possibility that glutamine deprivation might increase LDH leakage because there was more intracellular LDH, if glutamine-deprived cells used lactate as an alternative metabolic substrate to glutamine. However, LDH activity in cell lysates was similar after 24 h culture of cells with or without glutamine (Fig 3A; independent t-test, P = 0.65, n = 4 animals). To test the possibility that glutamine might bind to pyolysin or neutralize the activity of pyolysin, we used horse red blood cells, which are highly sensitive to hemolysis caused by cholesterol-dependent cytolysins [14]. However, incubating glutamine with pyolysin did not affect hemolysis, whereas incubating pyolysin with cholesterol, as a control, reduced hemolysis, as expected for a cholesterol-dependent cytolysin (Fig 3B).

Alanine is an abundant non-essential amino acid in blood, and glutamine metabolism can also yield alanine [21, 31]. Therefore, we considered if alanine might mimic the cytoprotective effect of glutamine. Using glutamine-free medium, we cultured bovine endometrial stromal cells with or without 2 mM alanine for 24 h prior to challenge with a range of concentrations of pyolysin. However, supplying alanine did not significantly protect the cells from the leakage of LDH after a 2 h challenge with PLO, compared with cells cultured without alanine (Fig 3D).

As supplying glutamine prior to pyolysin challenge supported cytoprotection, we wondered whether glutamine could also help cells recover after pyolysin challenge. Cells were cultured for 24 h in the presence or absence of 2 mM glutamine and then challenged for 2 h with pyolysin, after which the media were replenished with or without 2 mM glutamine for a further 24 h. Cellular DNA was measured at the end of the experiment to estimate cell survival. Cells treated with glutamine prior to pyolysin challenge showed no significant difference (P = 0.18) in cellular DNA remaining when media were replenished with glutamine or not after pyolysin challenge (Fig 3C, 2–2 and 2–0), with cell survival reduced by 26% and 33%, respectively. However, deprivation of glutamine prior to pyolysin challenge, irrespective of whether media were replenished with glutamine or not after pyolysin challenge, reduced cell survival by 70% and 88%, respectively (Fig 3C, 0–2 and 0–0, P < 0.001). These data provide evidence that glutamine supported cytoprotection against a pyolysin challenge, but glutamine did not help recovery after a pyolysin challenge.

### Glutamine supports stromal cell protection against streptolysin O

**Fig 4:**
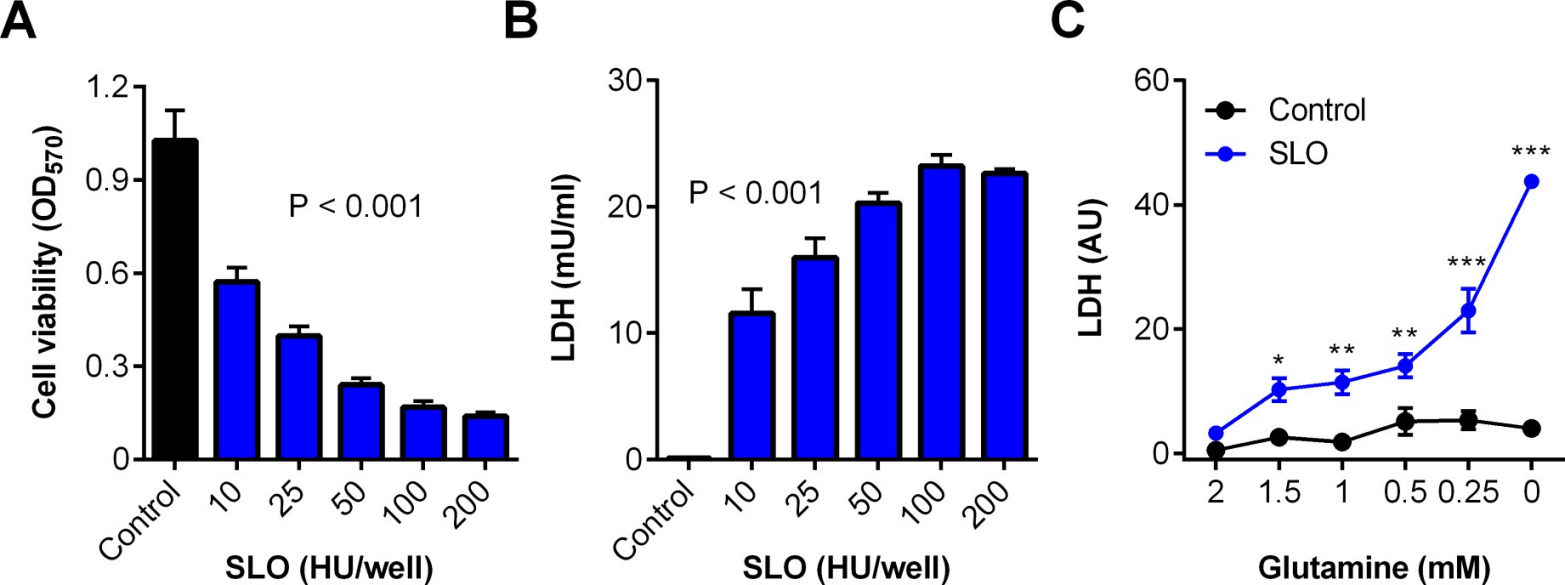
Glutamine is cytoprotective against streptolysin O in stromal cells. Bovine endometrial stromal cells were challenged for 2 h with control serum-free medium (∎) or medium containing the indicated concentrations of SLO (∎); cell viability was determined by MTT assay (A) and LDH leakage measured in the cell supernatants (B). Data are presented as mean (SEM) using cells isolated from 4 animals; data were analyzed by ANOVA and P values reported. (C) Cells were cultured in medium containing the indicated concentrations of glutamine for 24 h, and challenged for 2 h with control medium (•) or 10 HU SLO (•), and LDH measured in supernatants and normalized to cellular DNA in the control challenge. Data are mean (SEM) of cells isolated from 4 animals, analyzed by ANOVA with Dunnett’s post hoc test; values differ from 2 mM glutamine SLO challenge, * P < 0.05, ** P < 0.01, *** P <0.001.

We next examined whether glutamine affected stromal cytoprotection against another cholesterol-dependent cytolysin, streptolysin O (SLO). We first determined that a 2 h challenge with SLO caused cell damage to bovine endometrial stromal cells, as determined by reduced cell viability and leakage of LDH (Fig 4A and 4B). However, limiting the availability of glutamine increased the leakage of LDH into cell supernatants when cells were challenged with SLO (Fig 4C; two-way ANOVA; n = 4 animals, P < 0.001). Together the data from Figs 2 to 4 provide evidence that glutamine supports stromal cell protection against cholesterol-dependent cytolysins.

### Glutamine supports HeLa cell protection against pyolysin and streptolysin O

**Fig 5:**
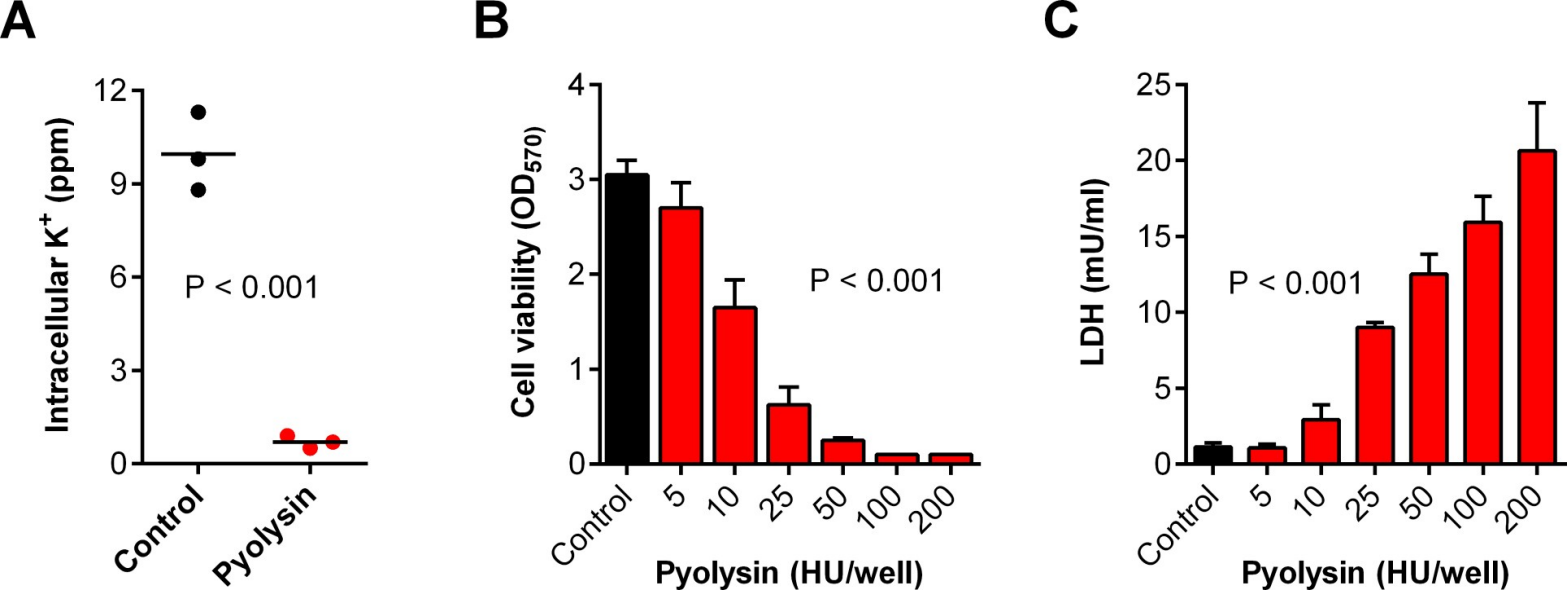
Cytolytic activity of pyolysin in HeLa cells. (A) HeLa cells were challenged for 5 min with control serum-free medium (•) or medium containing pyolysin (•), and potassium was measured in cell lysates. Data are presented using cells from 3 independent cell passages and the horizontal line represents the mean; data were analyzed by t-test. (B, C) Cells were challenged for 2 h with control serum-free medium (∎) or medium containing the indicated concentrations of pyolysin (∎); cell viability was determined by MTT assay and LDH leakage measured in the cell supernatants. Data are presented as mean (SEM) using cells from 4 passages; data were analyzed by ANOVA and P values reported.

We next examined whether glutamine cytoprotection against cholesterol-dependent cytolysins was restricted to bovine endometrial stromal cells. We used immortalized human cervical epithelial cells, HeLa cells, because they are widely employed to examine tissue cell responses to cholesterol-dependent cytolysins [6, 32, 33]. First, we established that challenging HeLa cells with pyolysin caused pore-formation, as determined by a reduction in intracellular potassium after 5 min (Fig 5A), a reduction in cell viability after 2 h (Fig 5B), and an increase in the leakage of LDH from the cytosol into cell supernatants (Fig 5C).

**Fig 6:**
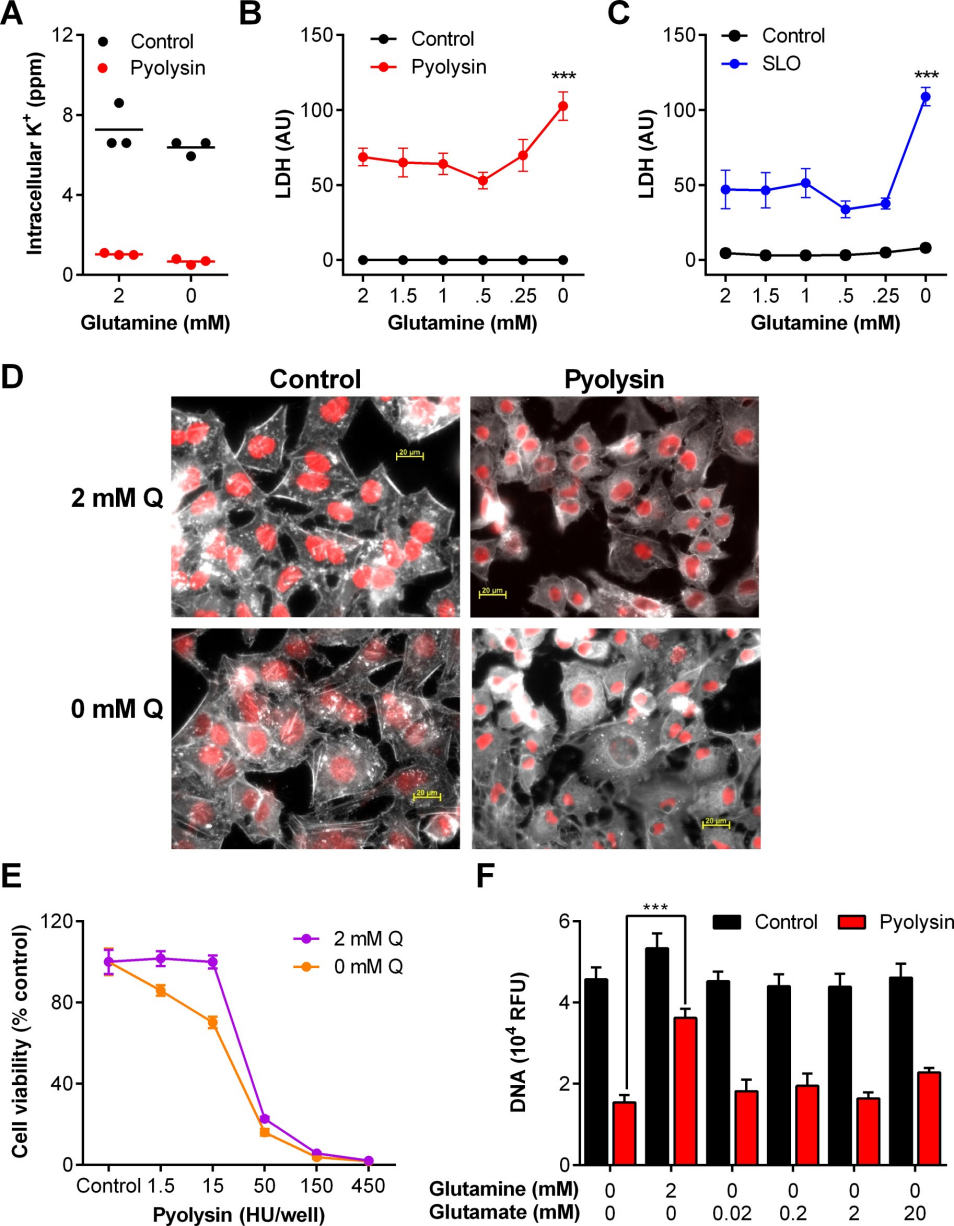
Glutamine is cytoprotective against pyolysin in HeLa cells. (A) HeLa cells were cultured for 24 h in medium containing 2 mM glutamine or without glutamine, and challenged for 5 min with control medium (•) or pyolysin (•). Intracellular potassium was determined by flame photometry. Data are from 3 independent passages, with the horizontal line indicating the mean. (B, C) Cells were cultured in medium containing the indicated concentrations of glutamine for 24 h, and challenged for 2 h with control medium (•), 10 HU pyolysin (•) or 10 HU SLO (•), and LDH measured in supernatants and normalized to cellular DNA in the control challenge. Data are mean (SEM) of 4 passages, analyzed by ANOVA with Dunnett’s post hoc test; values differ from 2 mM glutamine cytolysin challenge, *** P <0.001. (D) Cells were cultured for 24 h in the presence of 2 mM glutamine (2 mM Q) or without glutamine (0 mM Q) in serum-free media, and then challenged for 2 h with control medium or pyolysin. The cells were stained with Alexa Fluor 555-conjugated phalloidin to visualize F-actin (white) and fluorescent microscope images collected; nuclei are red; scale bars are 20 μm. Images are representative of 3 experiments. (E) Cells were treated with medium, containing 10% fetal bovine serum, with glutamine (2 mM Q, •) or without glutamine (0 mM Q, •) for 24 h before challenge with the indicated concentrations of pyolysin. Cell viability was determined by MTT assay and expressed as the percent of control. Data are mean (SEM) of 4 passages. (F) Cells were cultured in serum-free medium with the indicated concentrations of glutamine or glutamate for 24 h before challenge with control medium (.) or pyolysin (∎) for 2 h and cellular DNA measured. Data are mean (SEM) from 6 independent passages, and analyzed by ANOVA and Bonferroni post hoc test, *** P <0.001.

To examine if the availability of glutamine affected the protection of HeLa cells against pyolysin, HeLa cells were cultured for 24 h in serum-free media containing excess glucose (25 mM) with a range of concentrations of glutamine (0 to 2 mM), and then challenged for 2 h with control medium or 10 HU/well pyolysin. Irrespective of glutamine availability, pyolysin caused pore formation, as determined by loss of intracellular potassium within 5 min (Fig 6A; two-way ANOVA, P < 0.001). However, limiting the availability of glutamine increased the accumulation of LDH in supernatants when HeLa cells were challenge with pyolysin (Fig 6B; two-way ANOVA, P < 0.001). HeLa cells are also sensitive to SLO [33, 34], and we found that glutamine deprivation also increased LDH leakage when HeLa cells were challenges with SLO (Fig 6C; two-way ANOVA, P < 0.001). Staining actin with Alexa Fluor 555-conjugated phalloidin also showed that HeLa cells cultured without glutamine and challenged with pyolysin had a diffuse actin cytoskeleton, presence of vacuoles, and disruption of the cell boundaries with membrane blebs. Whereas, when cells were cultured with glutamine and challenged with pyolysin, even though they lost their angular shape, the cells usually maintained defined cell boundaries (Fig 6D).

We further validated the cytoprotective effect of glutamine in HeLa cells by taking advantage of similar growth curves for HeLa cells irrespective of the glutamine supply when cells were cultured with 10% fetal bovine serum, as determined by MTT assay (S1 Fig). Cells cultured with glutamine prior to pyolysin challenge were less sensitive to cytolysis than cells cultured in 2 mM glutamine (Fig 6E; two-way ANOVA, P = 0.001).

One consideration was whether cytoprotection was specific to glutamine, or whether the cytoprotective effect of glutamine could be mimicked using the glutamine derivative, glutamate, which is another non-essential amino acid [21, 31]. Using glutamine-free medium, we cultured HeLa cells with or without a range of concentrations of glutamate for 24 h prior to challenge with pyolysin. Cellular DNA was measured at the end of the experiment to estimate cell survival. Cells treated with glutamine prior to pyolysin challenge protected against cytolysis compared with cells without glutamine or glutamate (Fig 6F; 68% vs 34% survival). However, supplying glutamate did not significantly alter the leakage of LDH after a 2 h challenge with PLO, compared with cells cultured without glutamate (Fig 6F; 37% survival with 2 mM glutamate). In summary, the data in Fig 6 provide evidence that glutamine supports HeLa cell protection against cholesterol-dependent cytolysins.

### Glutaminolysis was not essential for cytoprotection against pyolysin

**Fig 7:**
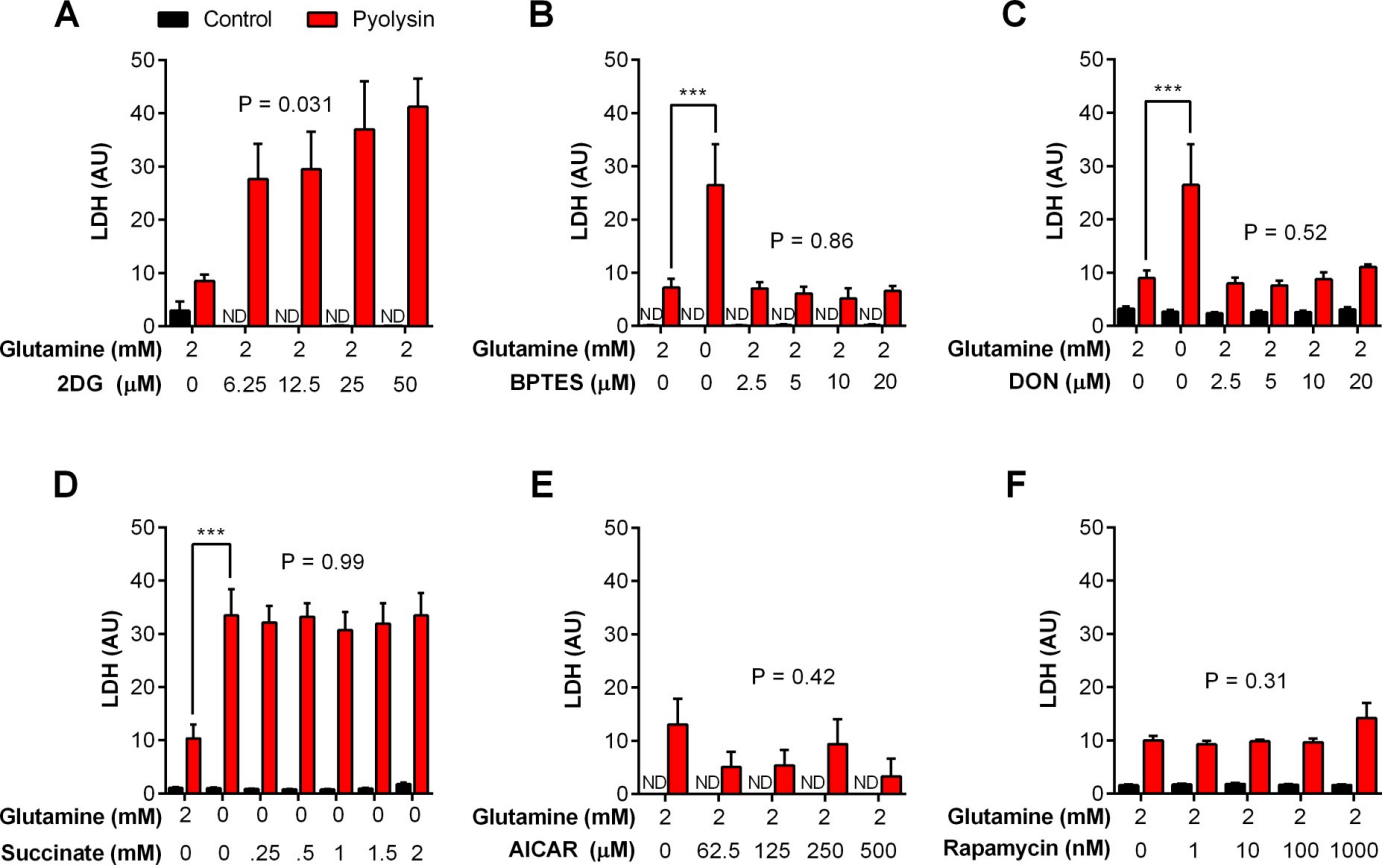
Stromal cell metabolism and cytoprotection against pyolysin. Bovine endometrial stromal cells were cultured in serum-free media for 24 h with the indicated concentrations of glutamine and the glycolysis inhibitor 2-deoxy-D-glucose (2DG; A), glutaminolysis inhibitors BPTES (B) or DON (C), succinate (D), AMPK activator AICAR (E), or mTOR inhibitor rapamycin (F), and then challenged for 2 h with control medium (∎) or pyolysin (∎). The leakage of LDH from cells was measured in cell supernatants, and normalized to cellular DNA in the control challenge. Data are presented as mean (SEM) using cells isolated from 4 animals for each experiment. Data were analyzed by ANOVA and Dunnett’s post hoc test; the reported P values are the effect of the treatment on the response to pyolysin challenge. ND, not detectable.

One obvious potential mechanism for the glutamine cytoprotection against cholesterol-dependent cytolysins is that glutamine could supply cellular energy—even though the cells were supplied with excess glucose (11 mM for stroma, 25 mM for HeLa cells). First we showed that glucose was used by the cells for energy because inhibiting glycolysis, using 2-deoxy-D-glucose [9], markedly increased LDH leakage from stromal cells challenged with pyolysin, when cells were supplied with 2 mM glutamine (Fig 7A).

To explore the importance of glutamine as an energy substrate for cytoprotection, we examined the role of glutaminolysis, whereby glutaminase converts glutamine to glutamate, which is metabolized to succinate to replenish the Krebs cycle [9, 24]. To inhibit glutaminolysis we used the inhibitor BPTES, a bis-thiadiazole that induces an inactive conformation of glutaminase [35, 36], and DON, a non-standard amino acid 6-Diazo-5-oxo-L-norleucine that covalently binds glutaminase [37, 38]. We postulated that inhibiting glutaminase in cells supplied with 2 mM glutamine would mimic glutamine deprivation, leading to increased leakage of LDH. As before, in the absence of the glutaminase inhibitors, glutamine deprivation increased the leakage of LDH from cells challenged with pyolysin (Fig 6B and 6C). However, the leakage of LDH after pyolysin challenge was not significantly increased in cells treated with BPTES (Fig 7B; two-way ANOVA, P = 0.86) or DON (Fig 7C; two-way ANOVA, P = 0.52). In a complementary approach, we cultured cells with a range of concentrations of succinate, in glutamine-free medium, to assess whether the beneficial effect of glutamine was by replenishing the Krebs cycle, and to account for the GABA (gamma-aminobutyric acid) shunt converting glutamine to succinate [9]. However, supplying succinate did not significantly reduce the leakage of LDH from cells in glutamine-free medium (Fig 7D; two-way ANOVA, P = 0.99).

Cells regulate their energy homeostasis using AMP-activated protein kinase (AMPK), which senses increased AMP:ATP ratios, and mammalian target of rapamycin (mTOR), which integrates satiety signals from hormones, growth factors, and the abundance of amino acids, including glutamine [39, 40]. As glutamine influences AMPK and mTOR signaling [39, 41], we considered whether AMPK and mTOR might affect the ability of glutamine to support cytoprotection against pyolysin. However, there was no substantive change in LDH leakage from cells challenged with pyolysin when mimicking metabolic energy deficits by activating AMPK with AICAR (Fig 7E; ANOVA, P = 0.42) or inhibiting mTOR with rapamycin (Fig 6F: ANOVA, P = 0.31). Together, the data in Fig 7 provide evidence that glutamine supports cytoprotection against pyolysin but that this cytoprotection was not dependent on glutamine replenishing the Krebs cycle.

### Glutamine and cellular cholesterol

**Fig 8:**
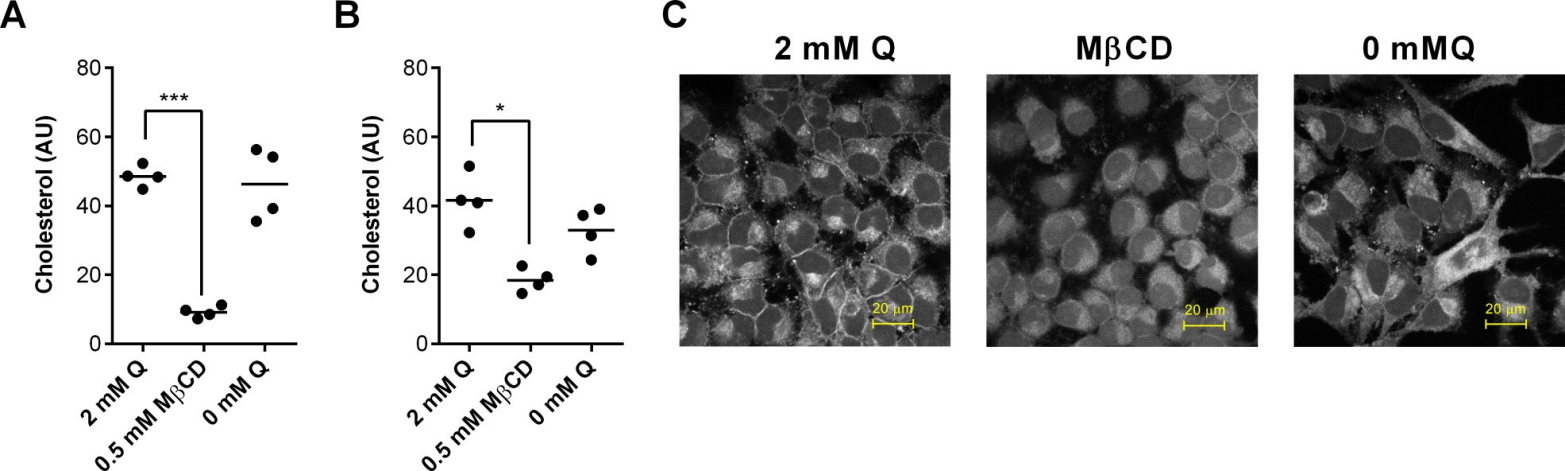
Glutamine and cellular cholesterol. HeLa cells (A) and bovine endometrial stromal cells (B) were cultured for 24 h in serum-free media with glutamine (2 mM Q), 0.5 mM methyl-β-cyclodextrin (MβCD), or without glutamine (0 mM Q). Cellular cholesterol was measured and normalized to the phospholipid content to account for differences in cell growth. Data are presented using 4 independent cell passages for HeLa cells or 4 animals for stromal cells; the horizontal line represents the mean. Data were analyzed by ANOVA and Dunnett’s post hoc test; values differ from 2 mM glutamine, * P < 0.05, *** P < 0.001. (C) Confocal microscope images of HeLa cells stained with filipin to visualize cholesterol (white) for the indicated treatments; scale bars are 20 μm.

A second potential mechanism for glutamine cytoprotection against cholesterol-dependent cytolysins is that glutamine could reduce cellular cholesterol. Methyl-β-cyclodextrin is widely used to reduce cellular cholesterol [14, 32], and in the present study, treating cells with 0.5 mM methyl-β-cyclodextrin for 24 h also reduced cellular cholesterol in HeLa cells (Fig 8A) and in stromal cells (Fig 8B). This reduced cellular cholesterol also protected the cells against a 2 h pyolysin challenge, as determined by MTT assay for HeLa cells (methyl-β-cyclodextrin vs. vehicle, 96.6 ± 7.3 vs. 14.8 ± 0.5% viability of control; P < 0.001, t-test, n = 4) and stromal cells (methyl-β-cyclodextrin vs. vehicle, 76.7 ± 13.4 vs. 11.8 ± 3.2% viability of control; P < 0.001, t-test, n = 7). However, cholesterol concentrations did not significantly differ between cells cultured with or without 2 mM glutamine for both HeLa cells (Fig 8A, P = 0.63) or endometrial stromal cell (Fig 8B, P = 0.26). We also took advantage of the well-defined HeLa cell shape and used filipin and confocal microscopy to examine the distribution of cholesterol [42]. HeLa cells cultured with or without glutamine showed little difference in staining intensity (Fig 8C). Taken together these data do not support the idea that glutamine could alter cytoprotection against cholesterol-dependent cytolysins by modifying the abundance of cellular cholesterol.

## Discussion

We found that glutamine supports the protection of tissue cells against the damage caused by cholesterol-dependent cytolysins from pathogenic bacteria. The role of glutamine in cytoprotection was unexpected, but was consistent across a range of experiments. Glutamine supported cytoprotection against two different cholesterol-dependent cytolysins, pyolysin and streptolysin O, in two disparate tissue cell types, primary bovine endometrial stromal cells and immortalized human cervical epithelial cells.

Supplying glutamine reduced the damage that the cytolysins caused to the cells, even though pores formed in the cell membrane, as evidenced by the rapid leakage of potassium from cells. This glutamine cytoprotection was evident by examining cell viability, imaging cells, and by measuring the leakage of LDH from cells. We chose a 2 h challenge with cholesterol-dependent cytolysins to test cytoprotection, rather than a longer cytolysin challenge, which might also reflect longer-term immune responses, or damage repair and recovery responses [6, 30]. Finding that glutamine reduced pyolysin-induced cell death if given before, but not after pyolysin challenge also supports a role for glutamine in protecting cells, rather than damage repair. Glutamine was specifically required for cytoprotection as glutamate or alanine did not mimic the cytoprotection against pyolysin provided by glutamine.

Infections are metabolically demanding [9, 25, 43]. We therefore considered whether cytoprotection against cholesterol-dependent cytolysins might depend on glutamine replenishing the Krebs cycle [9]. Glutaminase is active in fibroblasts and HeLa cells, converting glutamine to glutamate, which is then used to yield succinate for the Krebs cycle [44, 45]. However, inhibitors of glutaminase did not impair cytoprotection against pyolysin in the present study. Furthermore, supplying succinate to cells in glutamine-free media did not enhance cytoprotection. Damage and infections also activate AMPK, and glutamine regulates AMPK and mTORC1 signaling [39, 41], but manipulating AMPK or mTOR in the present study did not affect cytoprotection against pyolysin. Taken together, these lines of evidence imply that whilst glutamine was important for cytoprotection against cholesterol-dependent cytolysins, the mechanism did not depend on glutaminolysis. These findings are intriguing because although immunity and tolerance complement each other, they can employ different mechanisms [46–48]. Indeed, whilst succinate did not contribute to cytoprotection against cytolysins here, cellular succinate regulates innate immunity and drives inflammatory responses to bacterial lipopolysaccharide in macrophages [9, 10]. Furthermore, manipulating AMPK or mTOR, as well as reducing the availability of glucose or glutamine, impairs inflammatory responses to lipopolysaccharide in the bovine endometrium [49, 50].

Cholesterol is the binding target for pyolysin and SLO [5], and reducing cholesterol in the cell membrane increases cytoprotection against cholesterol-dependent cytolysins [14, 33, 51]. However, in the present study, any effects of glutamine on cellular cholesterol abundance or distribution were modest compared with methyl-β-cyclodextrin. These findings are plausible because glutamine can provide citrate for cholesterol synthesis, and glutamine stimulates the expression of genes associated with cholesterol synthesis [31, 52].

In the present *in vitro* studies, only 0.25 mM glutamine was needed to protect cells against the cytolysins, and there is about 0.7 mM glutamine in human plasma and 0.25 mM in bovine plasma [8, 25]. However, the glutamine available for cellular metabolism may be far less than implied by the plasma concentrations of glutamine. The availability of glutamine also depends on the rate of glutamine transporter-mediated influx and efflux, as well as glutamine synthesis [53, 54]. Glutamine even becomes conditionally essential during injury or infection, and glutamine supplementation is used therapeutically [8, 26, 27, 53]. Part of this increased demand for glutamine is to fuel the repair of tissue damage and the synthesis of inflammatory mediators. However, damage and inflammation may also limit the ability of glutamine to diffuse from blood vessels into the tissues. Furthermore, pathogenic bacteria, such as *Escherichia coli*, *Salmonella typhimurium* and *Streptococcus pneumoniae*, can take up glutamine or synthesize glutamine, and glutamine is important for their metabolism and virulence [55–57]. To explore the integration of glutamine physiology between cells and bacteria, future studies could manipulate the supply of glutamine to stromal and epithelial cells challenged with live *T*. *pyogenes* and beta-hemolytic group A *Streptococci*. However, this will be challenging as the mammalian cells and bacteria differ in their culture conditions *in vitro*. As glutamine has multiple roles during infections, and contributes to amino acids, proteins, and nucleotides as well as supplying the Krebs cycle, future studies might also use radiolabeled glutamine to trace how glutamine contributes to cytoprotection and bacterial virulence.

Cell protective mechanisms against pore-forming toxins include changes in the plasma membrane and membrane receptors, activation of cell stress responses, membrane repair, and cytoskeletal maintenance [6, 7, 15, 30, 32, 34, 58]. Cell stress responses and membrane repair in response to damage caused by cholesterol-dependent cytolysins has been studied widely, but not explicitly linked to the supply of glutamine. An interesting feature noted in the present study was that glutamine was important to limit toxin-induced changes in the actin cytoskeleton and plasma membrane, which might help cells tolerate damage, changes in osmotic pressure, or the remodeling of actin during pore-formation [59]. Another aspect that might be explored in the future is a link between glutamine and epidermal growth factor receptor function, which protects the gut barrier and tight junctions between epithelial cells against damage caused by alcohol [60]. The beneficial role of glutamine in tissue cytoprotection complements the role glutamine plays in immune cell metabolism and supporting inflammatory responses to pathogens [9, 48]. However, it is unlikely that the glutamine cytoprotection was dependent on cytokines released from cells during the experiments because bovine endometrial stromal cells and peripheral blood mononuclear cells do not secrete interleukins in response to pyolysin [14].

In conclusion, we found that glutamine supports the protection of tissue cells against the damage caused by cholesterol-dependent cytolysins from pathogenic bacteria. More work will be needed to determine the mechanism linking glutamine to cytoprotection against cytolysins. However, the implication of finding that glutamine supports cytoprotection is that glutamine may help tissues to tolerate pathogenic bacteria that secrete cholesterol-dependent cytolysins.

## Methods

### Ethical statement

No live animal experiments were performed. Uteri were collected from cattle after slaughter and processing, as part of the normal work of a commercial slaughterhouse, with approval (registration number U1268379/ABP/OTHER) from the United Kingdom (UK) Department for Environment, Food and Rural Affairs under the Animal By-products Registration (EC) No. 1069/2009.

### Cell culture

To isolate primary bovine endometrial stromal cells, uteri were collected after slaughter from post pubertal, non-pregnant animals with no evidence of genital disease or microbial infection. Endometrial stromal cells were isolated, cell purity confirmed, and the absence of immune cell contamination verified, as described previously [14, 28, 29]. A standard operating procedure details the isolation of these primary cells from bovine uteri [61]. Briefly, stromal cells were isolated by enzymatic digestion of the endometrium, sieving the cell suspension through a 70-μm mesh to remove debris, and then through a 40-μm mesh (pluriStrainer®, Cambridge Bioscience, Cambridge, UK) to isolate stromal cells, followed by adhesion to culture plates within 18 h, at which time any contaminating epithelial cells were washed away. The cells were maintained in 75 cm^2^ flasks (Greiner Bio-One, Gloucester, UK) with complete medium, comprising RPMI-1640 medium (Catalogue number #61870, Thermo Fisher Scientific, Paisley, UK), 10% fetal bovine serum (Biosera, East Sussex, UK), 50 IU/ml of penicillin, 50 μg/ml of streptomycin and 2.5 μg/ml of amphotericin B (all Sigma, Gillingham, UK). Cells were used from passage 1 and 2.

The immortal HeLa cells were purchased for the study (Public Health England, Salisbury, UK; #93021013, HeLa CCL2), and cultured cells were maintained in 75 cm^2^ flasks with complete medium, comprising DMEM (#41965, Thermo Fisher Scientific) 10% fetal bovine serum, 50 IU/ml penicillin, 50 μg/ml streptomycin, and 2.5 μg/ml amphotericin B. Cells were used from passage 5 to 25. The HeLa cell identity was confirmed at the end of the study by short tandem repeat profiling (Report Reference number SOJ39361; ATCC, Manassas, VA, USA). Cells were incubated at 37°C in humidified air with 5% CO_2_.

### Cholesterol-dependent cytolysins

The *plo* plasmid (pGS59) was a gift from Dr H Jost (University of Arizona), and pyolysin protein was generated as described previously [14, 62]. The activity of pyolysin was 628,338 HU/mg protein, as determined by hemolysis assay using horse red blood cells (Oxoid, Hampshire, UK), as described previously [14, 63]. Endotoxin contamination was 1.5 EU/mg protein, as determined by a limulus amebocyte lysate assay (LAL endotoxin quantitation kit; Thermo Fisher Scientific, Hertfordshire, UK). Streptolysin O was purchased from Sigma, stored as 1 mg/ml solution, and activated with 10 mM dithiothreitol according to the manufacturer’s instructions (Sigma). To examine the potential for pyolysin binding to glutamine, 100 HU/ml pyolysin was incubated for 1 h in PBS with vehicle, 2 mM glutamine, or 1 mM cholesterol as a positive control, and a hemolysis assay was conducted.

### Glutamine manipulation

The bovine endometrial stromal cells were seeded at 5 × 10^4^ cells/well in 24-well plates and incubated for 24 h in complete medium. The cells were then incubated for 24 h in serum-free medium containing the amounts of L-glutamine specified in *Results*, which were generated by combining defined ratios of RPMI1640 with or without L-glutamine (#11875, 11.1 mM glucose, 2 mM glutamine; and, #31870, 11.1 mM glucose, no glutamine; Thermo Fisher Scientific).

The HeLa cells were seeded at 4 × 10^4^ cells/well in complete medium for 24 h, followed by a further 24 h in serum-free medium containing the amounts of L-glutamine specified in *Results*, which were generated by combining defined ratios of DMEM with or without L-glutamine (#41965, 25 mM glucose, 4 mM glutamine; and, #11960, 25 mM glucose, no glutamine; Thermo Fisher Scientific).

After the treatment period, cells were challenged with their corresponding control medium, or medium containing pyolysin or SLO, as specified in *Results*. In some experiments, transmitted light images of the cells were collected using an Axiovert 40C inverted microscope and AxioCam ERc5s camera (Zeiss, Jena, Germany). At the end of experiments, cell supernatants were collected for LDH quantification, and cells used for measuring cellular DNA or viability.

### Nutrients and inhibitors

To examine the effect of nutrients, cells were seeded and cultured for 24 h in complete media, and then cultured for 24 h in serum-free media containing the amounts reported in *Results* of L-alanine (#05129, Sigma), glutamate (L-Glutamic acid monosodium salt hydrate, #G5889, Sigma) or dimethyl succinate (#W239607, Sigma). After 24 h treatment, cells were challenged for 2 h with pyolysin, and supernatants and cells collected. To examine the effect of inhibitors, cells were seeded and cultured for 24 h in complete media, and then cultured for 24 h in serum-free media containing the amounts reported in *Results* of the glycolysis inhibitor 2-deoxy-D-glucose (2DG; #D3179, Sigma), the glutaminase inhibitors BPTES (#314045, EMD Millipore, Hertfordshire, UK) or DON (#D2141, Sigma), the AMPK activator AICAR (#2840, Tocris, Bristol, UK), the mTOR inhibitor rapamycin (#553211, EMD Millipore), or methyl-β-cyclodextrin (#332615, Sigma). After 24 h treatment, cells were challenged for 2 h with control medium or pyolysin, and supernatants and cells collected.

### Cell viability

The mitochondria-dependent reduction of 3-(4,5-dimethylthiazol-2-yl)-2,5-diphenyltetrazolium bromide (MTT, Sigma) to formazan was used to assess cell viability, as described previously [14]. As nutrient availability may influence the reduction of MTT, cell abundance was also determined by measuring cellular DNA content. Briefly, at the end of experiments when supernatants were removed, the cells were washed in 500 μl ice-cold PBS before being stored at -80°C overnight to ensure lysis, and DNA was measured using the CyQUANT Cell Proliferation Assay Kit (Thermo Fisher Scientific).

### Lactate dehydrogenase and potassium leakage

Lactate dehydrogenase leakage from cells was measured in cell supernatants using a Lactate Dehydrogenase Activity Assay Kit (Cambridge Bioscience) [6, 63]. Where indicated in *Results*, LDH leakage from cells was normalized to the cellular DNA in the control challenge.

To examine potassium leakage, 7.5 × 10^5^ cells were seeded in 75 cm^2^ culture flasks in complete media for 24 h, before treatment with or without 2 mM glutamine for a further 24 h in serum-free media. Media were then discarded and cells washed three times with potassium-free choline buffer (129 mM choline-Cl, 0.8 mM MgCl_2_, 1.5 mM CaCl_2_, 5 mM citric acid, 5.6 mM glucose, 10 mM NH_4_Cl, 5 mM H_3_PO_4_, pH 7.4; all Sigma). Cells were then incubated in choline-buffer with control medium or pyolysin for 5 min at 37°C. Subsequently, cells were washed three times in ice-cold choline-buffer and lysed in 0.5% Triton X-100 (Sigma) in double-distilled water for 20 min at room temperature with gentle agitation. Potassium was measured in the cleared lysates using a Jenway PFP7 flame photometer (Cole-Parmer, Stone, Staffordshire, UK).

### Cholesterol assay

The bovine endometrial stromal cells and HeLa cells were grown at a density of 10^5^ cells/well in 12-well tissue culture plates for 24 h in complete media, and then cultured for 24 h in serum-free media with or without 2 mM glutamine, or 0.5 mM methyl-β-cyclodextrin, as described in *Results*. After the treatment period, cells were collected in 200 μl/well cholesterol assay buffer (Thermo Fisher Scientific) and stored in Eppendorf tubes at -20°C. When needed, samples were defrosted at room temperature and sonicated for 10 min in a sonicating water bath. Cellular cholesterol content was measured using the Amplex® Red Cholesterol Assay Kit (Thermo Fisher Scientific). Total cellular phospholipid was measured in the samples prepared for the cholesterol assay using a phospholipid assay kit (MAK122, Sigma). Cholesterol concentrations were then normalized to phospholipid concentrations.

### Immunofluorescence

To examine actin distribution, cells were seeded at a 5 x 10^4^ cells on glass coverslips in a 24-well plate in complete medium for 24 h, followed by a further 24 h in serum-free medium with or without 2 mM glutamine. Cells were challenged for 2 h with the corresponding control medium or medium containing pyolysin, as specified in *Results*. Cells were washed with PBS, fixed with 4% paraformaldehyde, washed in PBS and then permeabilized in 0.2% Triton X-100. Cells were then blocked using 0.5% bovine serum albumin and 0.1% Triton X-100 in PBS, followed by incubation with Alexa Fluor 555-conjugated phalloidin (Thermo Fisher Scientific). Cells were washed in 0.1% Triton X-100 in PBS three times and mounted onto microscope slides, using 4′,6-diamidino-2-phenylindole (Vectashield with DAPI; Vector Laboratories Inc., Burlington, CA, USA) to visualize cell nuclei. Cell morphology and target localization were analyzed with an Axio Imager M1 upright fluorescence microscope (Zeiss, Jena, Germany) and images captured using an AxioCamMR3.

To image cholesterol, 5 x 10^4^ cells were seeded on glass coverslips in a 24 well plate in complete medium for 24 h, followed by 24 h in serum-free medium with or without L-glutamine, or 0.5 mM methyl-β-cyclodextrin, as described in *Results*. Coverslips were washed with PBS, fixed with 4% paraformaldehyde, and washed with PBS. Coverslips were then incubated for 45 min at room temperature with 50 μg/ml filipin III from *Streptomyces filipinensis* (Sigma). Cells were washed with PBS before being mounted using 2.5% Mowiol mounting medium containing 2.5% DABCO (1,4-diazabicyclo-(2,2,2)-octane, Merck). Cell cholesterol was analyzed using a LSM710 confocal microscope (Zeiss) with the Zeiss Zen 2010 software. Images were captured using a 40x oil objective and a 410–476 nm channel range; coverslips were subjected to identical exposure times and conditions.

### Statistical analysis

Data are presented as arithmetic mean and error bars represent SEM. The statistical unit was each animal used to isolate bovine endometrial stromal cells or each independent passage of HeLa cells. Statistical analysis was performed using SPSS 22.0 (SPSS Inc. Chicago, IL), and P < 0.05 was considered significant. Comparisons were made between treatments using one-way or two-way ANOVA with two-tailed Bonferroni or Dunnett’s posthoc test, or unpaired two-tailed Student’s t test, as specified in *Results* and figure legends.

## Supporting information

S1 Fig Similar cell growth curves irrespective of glutamine supply for HeLa cells cultured with serum. HeLa cells were cultured in medium containing 10% fetal calf serum and 2 mM glutamine for 24 h, and then with or without 2 mM glutamine for a further 72 h. Cell viability was measured using the MTT assay every 24 h. The data are reported as mean (SEM) from 4 independent passages. Data were analyzed by 2-way ANOVA; there was a significant effect of time (F_(3, 24)_ = 113.6, P < 0.0001) but not for glutamine (F_(1, 24)_ = 0.0005, P = 0.98) or the interaction of time x glutamine (F_(3, 24)_ = 0.5, P = 0.71).
(PDF)

## References

[1] Raberg L, Sim D, Read AF. Disentangling genetic variation for resistance and tolerance to infectious diseases in animals. Science. 2007;318(5851):812–4. Epub 2007/11/03. 10.1126/science.1148526 . 17975068

[2] Schneider DS, Ayres JS. Two ways to survive infection: what resistance and tolerance can teach us about treating infectious diseases. Nat Rev Immunol. 2008;8(11):889–95. Epub 2008/10/18. 10.1038/nri2432 18927577 PMC4368196

[3] Medzhitov R, Schneider DS, Soares MP. Disease tolerance as a defense strategy. Science. 2012;335(6071):936–41. 10.1126/science.1214935 22363001 PMC3564547

[4] Peraro MD, van der Goot FG. Pore-forming toxins: ancient, but never really out of fashion. Nat Rev Micro. 2016;14(2):77–92. 10.1038/nrmicro.2015.3 . 26639780

[5] Bischofberger M, Iacovache I, van der Goot FG. Pathogenic pore-forming proteins: function and host response. Cell Host Microbe. 2012;12(3):266–75. Epub 2012/09/18. 10.1016/j.chom.2012.08.005 . 22980324

[6] Gonzalez MR, Bischofberger M, Freche B, Ho S, Parton RG, van der Goot FG. Pore-forming toxins induce multiple cellular responses promoting survival. Cell Microbiol. 2011;13:1026–43. Epub 2011 Apr 26. 10.1111/j.1462-5822.2011.01600.x . 21518219

[7] Los FC, Randis TM, Aroian RV, Ratner AJ. Role of pore-forming toxins in bacterial infectious diseases. Microbiol Mol Biol Rev. 2013;77(2):173–207. Epub 2013/05/24. 10.1128/MMBR.00052-12 23699254 PMC3668673

[8] Curi R, Lagranha CJ, Doi SQ, Sellitti DF, Procopio J, Pithon-Curi TC, et al Molecular mechanisms of glutamine action. J Cell Physiol. 2005;204(2):392–401. Epub 2005/03/30. 10.1002/jcp.20339 . 15795900

[9] Tannahill GM, Curtis AM, Adamik J, Palsson-McDermott EM, McGettrick AF, Goel G, et al Succinate is an inflammatory signal that induces IL-1beta through HIF-1alpha. Nature. 2013;496(7444):238–42. Epub 2013/03/29. 10.1038/nature11986 . 23535595 PMC4031686

[10] Mills EL, Kelly B, Logan A, Costa ASH, Varma M, Bryant CE, et al Succinate Dehydrogenase supports metabolic repurposing of mitochondria to drive inflammatory macrophages. Cell. 2016;167(2):457–70. Epub 2016 Sep 22. 10.1016/j.cell.2016.08.064 27667687 PMC5863951

[11] Alouf JE. Streptococcal toxins (streptolysin O, streptolysin S, erythrogenic toxin). Pharmacol Ther. 1980;11(3):661–717. Epub 1980/01/01. 10.1016/0163-7258(80)90045-5 . 7003609

[12] Bhakdi S, Tranumjensen J, Sziegoleit A. Mechanism of membrane damage by streptolysin-O. Infect Immun. 1985;47(1):52–60. 3880730 10.1128/iai.47.1.52-60.1985 PMC261464

[13] Jost BH, Billington SJ. Arcanobacterium pyogenes: molecular pathogenesis of an animal opportunist. Antonie van Leeuwenhoek. 2005;88(2):87–102. 10.1007/s10482-005-2316-5 . 16096685

[14] Amos MR, Healey GD, Goldstone RJ, Mahan S, Duvel A, Schuberth HJ, et al Differential endometrial cell sensitivity to a cholesterol-dependent cytolysin links *Trueperella pyogenes* to uterine disease in cattle Biol Reprod. 2014;90:54 10.1095/biolreprod.113.115972 . 24478394

[15] Griffin S, Healey GD, Sheldon IM. Isoprenoids increase bovine endometrial stromal cell tolerance to the cholesterol-dependent cytolysin from *Trueperella pyogenes*. Biol Reprod. 2018;99(4):749–60. Epub 2018/04/25. 10.1093/biolre/ioy099 29688258 PMC6203874

[16] Hammon DS, Evjen IM, Dhiman TR, Goff JP, Walters JL. Neutrophil function and energy status in Holstein cows with uterine health disorders. Vet Immunol Immunopathol. 2006;113(1–2):21–9. Epub 2006 Jun 5. 10.1016/j.vetimm.2006.03.022 . 16740320

[17] LeBlanc SJ. Interactions of metabolism, inflammation, and reproductive tract health in the postpartum period in dairy cattle. Reprod Domest Anim. 2012;47 Suppl 5:18–30. Epub 2012/08/29. 10.1111/j.1439-0531.2012.02109.x . 22913557

[18] Yasui T, McCann K, Gilbert RO, Nydam DV, Overton TR. Associations of cytological endometritis with energy metabolism and inflammation during the periparturient period and early lactation in dairy cows. J Dairy Sci. 2014;97(5):2763–70. Epub 2014 Mar 5. 10.3168/jds.2013-7322 . 24612816

[19] Bromfield JJ, Santos JE, Block J, Williams RS, Sheldon IM. PHYSIOLOGY AND ENDOCRINOLOGY SYMPOSIUM: Uterine infection: linking infection and innate immunity with infertility in the high-producing dairy cow. J Anim Sci. 2015;93(5):2021–33. Epub 2015/05/29. 10.2527/jas.2014-8496 . 26020298

[20] Sheldon IM, Cronin JG, Bromfield JJ. Tolerance and innate immunity shape the development of postpartum uterine disease and the impact of endometritis in dairy cattle. Annu Rev Anim Biosci. 2019;7(1):361–84. Epub 2018 Oct 25. 10.1146/annurev-animal-020518-115227 30359085 PMC6450715

[21] DeBerardinis RJ, Mancuso A, Daikhin E, Nissim I, Yudkoff M, Wehrli S, et al Beyond aerobic glycolysis: Transformed cells can engage in glutamine metabolism that exceeds the requirement for protein and nucleotide synthesis. PNAS. 2007;104(49):19345–50. Epub 2007 Nov 21. 10.1073/pnas.0709747104 18032601 PMC2148292

[22] Lemons JM, Feng XJ, Bennett BD, Legesse-Miller A, Johnson EL, Raitman I, et al Quiescent fibroblasts exhibit high metabolic activity. PLoS Biol. 2010;8(10):e1000514 Epub 2010/11/05. 10.1371/journal.pbio.1000514 21049082 PMC2958657

[23] Reitzer LJ, Wice BM, Kennell D. Evidence that glutamine, not sugar, is the major energy source for cultured HeLa cells. J Biol Chem. 1979;254(8):2669–76. Epub 1979/04/25. . 429309

[24] Finley LW, Zhang J, Ye J, Ward PS, Thompson CB. SnapShot: cancer metabolism pathways. Cell Metab. 2013;17(3):466 e2 Epub 2013/03/12. 10.1016/j.cmet.2013.02.016 . 23473039

[25] Meijer GA, van der Meulen J, van Vuuren AM. Glutamine is a potentially limiting amino acid for milk production in dairy cows: a hypothesis. Metabolism. 1993;42(3):358–64. Epub 1993/03/01. 10.1016/0026-0495(93)90087-5 . 8487655

[26] Newsholme P. Why Is L-glutamine metabolism important to cells of the immune system in health, postinjury, surgery or infection? J Nutr. 2001;131(9):2515S–22S. 10.1093/jn/131.9.2515S . 11533304

[27] Wischmeyer PE, Kahana M, Wolfson R, Ren H, Musch MM, Chang EB. Glutamine reduces cytokine release, organ damage, and mortality in a rat model of endotoxemia. Shock. 2001;16(5):398–402. Epub 2001/11/09. 10.1097/00024382-200116050-00014 . 11699081

[28] Cronin JG, Turner ML, Goetze L, Bryant CE, Sheldon IM. Toll-Like receptor 4 and MyD88-dependent signaling mechanisms of the innate immune system are essential for the response to lipopolysaccharide by epithelial and stromal cells of the bovine endometrium. Biol Reprod. 2012;86:51 Epub 2011/11/05. 10.1095/biolreprod.111.092718 . 22053092 PMC4396703

[29] Turner ML, Cronin JC, Healey GD, Sheldon IM. Epithelial and stromal cells of bovine endometrium have roles in innate immunity and initiate inflammatory responses to bacterial lipopeptides in vitro via Toll-like receptors TLR2, TLR1 and TLR6. Endocrinology. 2014;155:1453–65. 10.1210/en.2013-1822 24437488 PMC3959608

[30] Witzenrath M, Pache F, Lorenz D, Koppe U, Gutbier B, Tabeling C, et al The NLRP3 Inflammasome Is Differentially Activated by Pneumolysin Variants and Contributes to Host Defense in Pneumococcal Pneumonia. J Immunol. 2011;187(1):434–40. 10.4049/jimmunol.1003143 . 21646297

[31] Zhang J, Pavlova NN, Thompson CB. Cancer cell metabolism: the essential role of the nonessential amino acid, glutamine. EMBO J. 2017;36(10):1302–15. Epub 2017/04/20. 10.15252/embj.201696151 . 28420743 PMC5430235

[32] Gurcel L, Abrami L, Girardin S, Tschopp J, van der Goot FG. Caspase-1 activation of lipid metabolic pathways in response to bacterial pore-forming toxins promotes cell survival. Cell. 2006;126(6):1135–45. 10.1016/j.cell.2006.07.033 . 16990137

[33] Preta G, Lotti V, Cronin JG, Sheldon IM. Protective role of the dynamin inhibitor Dynasore against the cholesterol-dependent cytolysin of *Trueperella pyogenes*. FASEB J. 2015;29(4):1516–28. Epub 2015/01/01. 10.1096/fj.14-265207 . 25550455 PMC4396600

[34] Idone V, Tam C, Goss JW, Toomre D, Pypaert M, Andrews NW. Repair of injured plasma membrane by rapid Ca2+-dependent endocytosis. J Cell Biol. 2008;180(5):905–14. Epub 2008/03/05. 10.1083/jcb.200708010 18316410 PMC2265401

[35] Robinson MM, McBryant SJ, Tsukamoto T, Rojas C, Ferraris DV, Hamilton SK, et al Novel mechanism of inhibition of rat kidney-type glutaminase by bis-2-(5-phenylacetamido-1,2,4-thiadiazol-2-yl)ethyl sulfide (BPTES). Biochem J. 2007;406(3):407–14. Epub 2007/06/22. 10.1042/BJ20070039 17581113 PMC2049044

[36] Thangavelu K, Pan CQ, Karlberg T, Balaji G, Uttamchandani M, Suresh V, et al Structural basis for the allosteric inhibitory mechanism of human kidney-type glutaminase (KGA) and its regulation by Raf-Mek-Erk signaling in cancer cell metabolism. PNAS. 2012;109(20):7705–10. Epub 2012 Apr 26. 10.1073/pnas.1116573109 22538822 PMC3356676

[37] Shapiro RA, Clark VM, Curthoys NP. Inactivation of rat renal phosphate-dependent glutaminase with 6-diazo-5-oxo-L-norleucine. Evidence for interaction at the glutamine binding site. J Biol Chem. 1979;254(8):2835–8. Epub 1979/04/25. . 429321

[38] Thangavelu K, Chong QY, Low BC, Sivaraman J. Structural basis for the active site inhibition mechanism of human kidney-type glutaminase (KGA). Sci Rep. 2014;4:3827 10.1038/srep03827 24451979 PMC4929687

[39] Hardie DG, Ross FA, Hawley SA. AMPK: a nutrient and energy sensor that maintains energy homeostasis. Nat Rev Mol Cell Biol. 2012;13(4):251–62. Epub 2012/03/23. 10.1038/nrm3311 . 22436748 PMC5726489

[40] Zoncu R, Efeyan A, Sabatini DM. mTOR: from growth signal integration to cancer, diabetes and ageing. Nat Rev Mol Cell Biol. 2011;12(1):21–35. Epub 2010/12/16. 10.1038/nrm3025 21157483 PMC3390257

[41] Jewell JL, Kim YC, Russell RC, Yu F-X, Park HW, Plouffe SW, et al Differential regulation of mTORC1 by leucine and glutamine. Science. 2015;347(6218):194–8. Epub 2015 Jan 7. 10.1126/science.1259472 25567907 PMC4384888

[42] Schwan C, Nolke T, Kruppke AS, Schubert DM, Lang AE, Aktories K. Cholesterol- and sphingolipid-rich microdomains are essential for microtubule-based membrane protrusions induced by Clostridium difficile transferase (CDT). J Biol Chem. 2011;286(33):29356–65. Epub 2011/06/28. 10.1074/jbc.M111.261925 21705797 PMC3190741

[43] Moret Y, Schmid-Hempel P. Survival for immunity: the price of immune system activation for bumblebee workers. Science. 2000;290(5494):1166–8. Epub 2000/11/10. 8972 [pii]. 10.1126/science.290.5494.1166 . 11073456

[44] Ghesquiere B, Wong BW, Kuchnio A, Carmeliet P. Metabolism of stromal and immune cells in health and disease. Nature. 2014;511(7508):167–76. Epub 2014/07/11. 10.1038/nature13312 . 25008522

[45] Nilsson R, Jain M. Simultaneous tracing of carbon and nitrogen isotopes in human cells. Mol Biosyst. 2016;12(6):1929–37. Epub 2016/04/22. 10.1039/c6mb00009f 27098229 PMC4879607

[46] McCarville JL, Ayres JS. Disease tolerance: concept and mechanisms. Curr Opin Immunol. 2017;50:88–93. Epub 2017/12/19. 10.1016/j.coi.2017.12.003 . 29253642 PMC5884632

[47] Soares MP, Teixeira L, Moita LF. Disease tolerance and immunity in host protection against infection. Nat Rev Immunol. 2017;17(2):83–96. Epub 2017/01/04. 10.1038/nri.2016.136 . 28044057

[48] Wang A, Luan HH, Medzhitov R. An evolutionary perspective on immunometabolism. Science. 2019;363(6423):eaar3932 Epub 2019/01/12. 10.1126/science.aar3932 30630899 PMC6892590

[49] Turner ML, Cronin JG, Noleto PG, Sheldon IM. Glucose availability and AMP-activated protein kinase link energy metabolism and innate immunity in the bovine endometrium. PLoS ONE. 2016;11:e0151416 Epub 2016/03/15. 10.1371/journal.pone.0151416 26974839 PMC4790959

[50] Noleto PG, Saut JP, Sheldon IM. Short communication: Glutamine modulates inflammatory responses to lipopolysaccharide in ex vivo bovine endometrium. J Dairy Sci. 2017;100(3):2207–12. Epub 2017/01/23. 10.3168/jds.2016-12023 . 28109606

[51] Giddings KS, Johnson AE, Tweten RK. Redefining cholesterol's role in the mechanism of the cholesterol-dependent cytolysins. PNAS. 2003;100(20):11315–220. Epub 2003/09/23. 10.1073/pnas.2033520100 14500900 PMC208754

[52] Inoue J, Ito Y, Shimada S, Satoh SI, Sasaki T, Hashidume T, et al Glutamine stimulates the gene expression and processing of sterol regulatory element binding proteins, thereby increasing the expression of their target genes. FEBS J. 2011;278(15):2739–50. Epub 2011/06/24. 10.1111/j.1742-4658.2011.08204.x . 21696544

[53] Cruzat V, Macedo Rogero M, Noel Keane K, Curi R, Newsholme P. Glutamine: metabolism and immune function, supplementation and clinical translation. Nutrients. 2018;10(11):1564 Epub 2018/10/27. 10.3390/nu10111564 30360490 PMC6266414

[54] Bhutia YD, Ganapathy V. Glutamine transporters in mammalian cells and their functions in physiology and cancer. Biochim Biophys Acta. 2016;1863(10):2531–9. Epub 2016/01/03. 10.1016/j.bbamcr.2015.12.017 26724577 PMC4919214

[55] Klose KE, Mekalanos JJ. Simultaneous prevention of glutamine synthesis and high-affinity transport attenuates Salmonella typhimurium virulence. Infect Immun. 1997;65(2):587–96. 9009317 10.1128/iai.65.2.587-596.1997 PMC176100

[56] Hendriksen WT, Kloosterman TG, Bootsma HJ, Estevão S, de Groot R, Kuipers OP, et al Site-specific contributions of glutamine-dependent regulator GlnR and GlnR-regulated genes to virulence of *Streptococcus pneumoniae*. Infect Immun. 2008;76(3):1230–8. Epub 2008 Jan 3. 10.1128/IAI.01004-07 18174343 PMC2258823

[57] Ma D, Lu P, Yan C, Fan C, Yin P, Wang J, et al Structure and mechanism of a glutamate–GABA antiporter. Nature. 2012;483(7391):632–6. 10.1038/nature10917 . 22407317

[58] Preta G, Jankunec M, Heinrich F, Griffin S, Sheldon IM, Valincius G. Tethered bilayer membranes as a complementary tool for functional and structural studies: The pyolysin case. Biochim Biophys Acta. 2016;1858(9):2070–80. Epub 2016/05/24. 10.1016/j.bbamem.2016.05.016 . 27211243

[59] Iliev AI, Djannatian JR, Nau R, Mitchell TJ, Wouters FS. Cholesterol-dependent actin remodeling via RhoA and Rac1 activation by the Streptococcus pneumoniae toxin pneumolysin. PNAS. 2007;104(8):2897–902. Epub 2007/02/16. 10.1073/pnas.0608213104 17301241 PMC1815278

[60] Meena AS, Shukla PK, Sheth P, Rao R. EGF receptor plays a role in the mechanism of glutamine-mediated prevention of alcohol-induced gut barrier dysfunction and liver injury. J Nutr Biochem. 2019;64:128–43. Epub 2018 Nov 6. 10.1016/j.jnutbio.2018.10.016 30502657 PMC6363835

[61] Cronin JG, Healey GD, Mackintosh SBP, Turner ML, Healy LL, Sheldon IM. Standard operating procedures for isolation and culture of primary bovine endometrial epithelial and stromal cells 2014; ResearchGate 10.13140/RG.2.2.29160.11529

[62] Billington SJ, Jost BH, Cuevas WA, Bright KR, Songer JG. The Arcanobacterium (Actinomyces) pyogenes hemolysin, pyolysin, is a novel member of the thiol-activated cytolysin family. J Bacteriol. 1997;179(19):6100–6. 10.1128/jb.179.19.6100-6106.1997 9324258 PMC179514

[63] Griffin S, Preta G, Sheldon IM. Inhibiting mevalonate pathway enzymes increases stromal cell resilience to a cholesterol-dependent cytolysin. Sci Rep. 2017;7(1):17050 10.1038/s41598-017-17138-y 29213055 PMC5719056

