# Supplementary material for: Glutamine supports the protection of tissue cells against the damage caused by cholesterol-dependent cytolysins from pathogenic bacteria
Source: PLoS One. 2020 Mar 12;15(3):e0219275. doi: 10.1371/journal.pone.0219275 (PMC7067430; doi:10.1371/journal.pone.0219275)
Supplement: S1 Fig — HeLa cells were cultured in medium containing 10% fetal calf serum and 2 mM glutamine for 24 h, and then with or without 2 mM glutamine for a further 72 h. Cell viability was measured using the MTT assay every 24 h. The data are reported as mean (SEM) from 4 independent passages. Data were analyzed by 2-way ANOVA; there was a significant effect of time (F(3, 24) = 113.6, P < 0.0001) but not for glutamine (F(1, 24) = 0.0005, P = 0.98) or the interaction of time x glutamine (F(3, 24) = 0.5, P = 0.71). (PDF) [file pone.0219275.s001.pdf]

S1 Fig

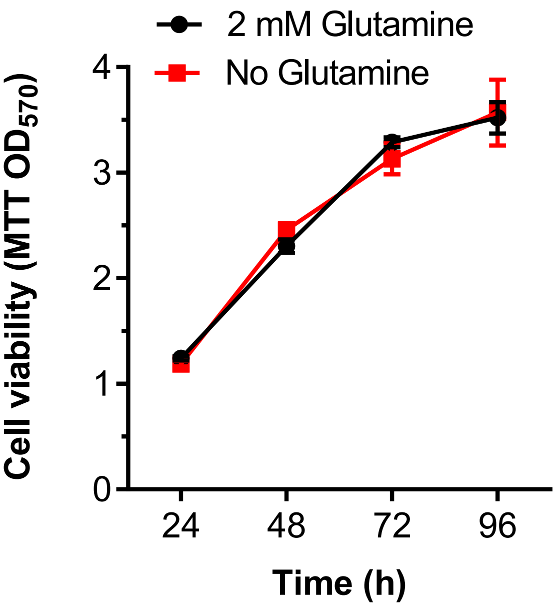

S1 Fig. Similar cell growth curves irrespective of glutamine supply for HeLa cells cultured with serum. HeLa cells were cultured in medium containing 10% fetal calf serum and 2 mM glutamine for 24 h, and then with or without glutamine for a further 72 h. Cell viability was measured using the MTT assay every 24 h. The data are reported as mean (SEM) from 4 independent passages. Data were analysed by 2-way ANOVA; there was a significant effect of time ($F_{(3, 24)} = 113.6$, $P < 0.0001$) but not for glutamine ($F_{(1, 24)} = 0.0005$, $P = 0.98$) or the interaction of time x glutamine ($F_{(3, 24)} = 0.5$, $P = 0.71$).
